# Case-Study-Based Overview of Methods and Technical Solutions of Analog and Digital Transmission in Measurement and Control Ship Systems

**DOI:** 10.3390/s22186931

**Published:** 2022-09-13

**Authors:** Mostafa Abotaleb, Janusz Mindykowski, Boleslaw Dudojc, Romuald Masnicki

**Affiliations:** 1 Doctoral School, Gdynia Maritime University, Morska 81-87, 81-225 Gdynia, Poland; 2 Faculty of Electrical Engineering, Gdynia Maritime University, Morska 81-87, 81-225 Gdynia, Poland

**Keywords:** Foundation Fieldbus, instrumentation amplifier, signal isolation, FISCO, FNICO, HPTC, DART, wireless HART, field barriers, segment protectors

## Abstract

The purpose of this article is to provide an overview of possible solutions to improve the performance of measurement and control processes in maritime engineering applications. This improvement can be basically provided by adopting techniques to enhance the reliability of measurement/control systems based on the 4–20 mA analogue standard. This aspect will be discussed through a Simscape Simulink model illustrating methods of noise and ground loops elimination for pressure measurement of a 4–20 mA current loop in the tank level measurement system on a bulk carrier commercial ship. Alternatively, improved measurement and control processes can be rendered by utilizing smart transmitters based on wired hybrid analogue–digital (Highway Addressable Remote Transducer (HART)), wired digital (Foundation Fieldbus (FF)) or wireless (wireless HART) communication protocols. A brief theoretical description of these protocols will be presented in this article. As an example of using smart transmitters, a simulation-based case study will analyze the possible options to implement non-intrinsically safe as well as intrinsically safe FF models for the tank level measurement system on a bulk carrier commercial ship. Conclusions obtained through analysis of the simulation results will characterize the behavior of FF segments in safe as well as explosive hazardous areas, highlighting the characteristics of field barriers and segment protectors used in conjunction with the HPTC (High-Power Trunk Concept) intrinsically safe model.

## 1. Introduction

Measurement and control systems on commercial ships are mostly similar to those existing in many land-based engineering applications, except for some systems that are particularly dedicated to maritime applications such as the scrubber system, sewage treatment plant system, oily water separator system, ballast water treatment system and inert gas system [1]. All these systems are extensively based on the 4–20 mA analogue standard due to its non-zero lower range limit and its high immunity to noise [1,2]. For the purpose of achieving the purpose of the article, which is describing the recommended methods to improve measurement/control processes on commercial ships, the tank level measurement system will be briefly described at the beginning of the article as an example for a maritime engineering application (based on the 4–20 mA analogue standard) on which the proposed methods can be applied. This description will be based on the associated problems of the system and their solution discussed in [2].

Based on the discussion in [2], which linked the 4–20 mA analogue standard to communication protocols that might coexist with it or replace it, the second section of the article will provide the basic knowledge needed for understanding technologies based on which smart sensors are constructed. These technologies are based on communication protocols such as HART, Foundation Fieldbus and wireless HART. Basic concepts of smart instrumentation as well as basic principles of technologies adopted by the most popular smart devices were explained in [3]. Basic principles of the HART protocol including frequency shift keying (FSK) modulation, communication modes and characteristics of the HART frame were explained in [2,4,5,6,7]. The improved performance rendered by HART transmitters can be manifested in features such as baseline estimation and loop characterization, the principles of which were presented in [8]. Similarly, main principles of Foundation Fieldbus such as basic concepts of communication technology (distributed data transfer DDT, scheduled/unscheduled communication and communication stack) adopted by the digital wired protocol, were presented in [2,9,10], ([11], pp. 21–55), [12,13], in addition to Manchester modulation/demodulation and noise elimination techniques [14]. Similar to baseline estimation and loop characterization in the HART protocol, increased reliability can be provided in Foundation Fieldbus transmitters by the feature of static process monitoring (SPM), which was explained in [15]. 

Other than wired digital smart transmitters, wireless digital smart transmitters such as wireless HART transmitters can be considered as an alternative for classical 4–20 mA analogue transmitters as well. The wireless HART protocol is based on time division multiple access (TDMA) for the purpose of scheduling communication [16,17,18] and the time synchronization mesh protocol for synchronized communication [16,18,19]. Wireless HART uses the direct sequence spread spectrum (DSSS) [20] in conjunction with the frequency-hopping spread spectrum (FHSS) [21]. Wireless HART has its own medium access control (MAC) sublayer [17,18] supervising the whole communication process, depending on the carrier sense multiple access with collision avoidance (CSMA-CA) technique [22,23,24]. The routing and scheduling algorithm adopted by wireless HART were explained in [25,26].

The contribution of this article is to discuss two recommended methods that can improve the performance of measurement/control systems on commercial ships (commonly based on the 4–20 mA standard). The first proposed method is based on the idea of reducing the possibility of ground loop formation through using signal isolators (optocoupler and transformer isolators) and elimination of common mode noise as well as coupled noise through the use of instrumentation amplifiers and low-pass filters, respectively. The second proposed method is to consider using smart transmitters, particularly Foundation Fieldbus transmitters, increasing the reliability of the measurement process. Both methods will be applied on seawater ballast tanks in a tank level measurement system. 

Application of the first method will be simulated through a Simulink Simscape model simulating how a 4–20 mA tank level measurement current loop can be integrated into an automation system. In ([27], pp. 335–338), discussion included the theoretical background for processes of amplification, isolation and analogue-to-digital conversion based on which the 4–20 mA analogue standard can be integrated in an automation system. Basic principles of optical and transformer isolation were provided in ([27], pp. 342–345, [28]). The optical isolation technique adopted by the model is based on using an expanded optocoupler model, as explained in [29]. The Simulink Simscape model includes a grounded load voltage-to-current converter, the basics of which are explained in [30,31]. Elimination of common mode noise and coupled noise was performed in the model using an instrumentation amplifier and low-pass filter, respectively. The basics of an instrumentation amplifier and low-pass filter were presented in [32,33].

Application of the second method will be rendered by Emerson Segment Design Tool (SDT) as well as Pepperl + Fuchs Segment Checker simulation models. These models will include a non-intrinsically safe model (safe area application) as well as five intrinsically safe models (hazardous area applications), which are the entity model, FISCO (Fieldbus Intrinsically Safe Concept), FNICO (Fieldbus Non-Incendive Concept), HPTC (High-Power Trunk Concept) and DART concept (Dynamic Arc Recognition and Termination). A general description of all FF intrinsically safe model was provided in [34,35]. Basic principles of the entity model were provided in [36]. Comparison between the entity and FISCO models and general features of the FISCO model were introduced in [37,38]. Basic principles of the FNICO model were explained in [39,40], while explanations for the HPTC model and DART model were presented in [41,42,43], respectively. 

Simulation results for both suggested methods, in addition to discussion and conclusions, will highlight the novelty elements introduced in this article. These novelty elements can be briefly described in two key points. The first key point is that simulation of the 4–20 mA analogue standard as an integrated part of an automation system rendered the adequate realization for the standard from a technical practical perspective linked to the application of the standard in maritime engineering (commercial ships), where additional preventive measures should be taken into account to overcome the negative effect induced by high levels of temperature, corrosion, humidity and salinity on the standard performance. The second key point is the detailed case-study-based comparative analysis conducted for FF models in safe areas as well as explosive hazardous areas, which identified in detail certain elements of novelty that characterize each FF model, particularly those related to the HPTC model and linked to the behavior of field barriers and segment protectors in HPTC segments, which can be the basis for future experimental research supported by additional simulation to estimate an equivalent mathematical model for the characteristics of field barriers and segment protectors.

## 2. Tank Level Measurement System (Problems and Recommended Solutions)

**Figure 1:**
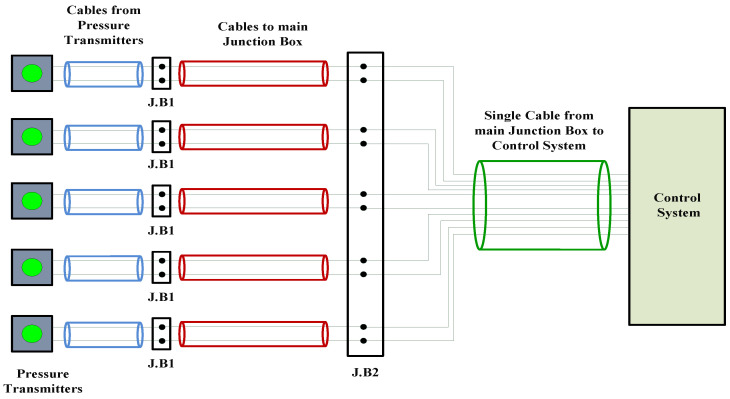
Connection diagram between 4–20 mA pressure transmitters and I/O modules in control system.

The tank level measurements system on any ship is responsible for measuring the fluid levels in all types of tanks on board. A brief description of the system was provided in [2]. There are many types of tanks on any commercial ship. These types are commonly similar in container ships and bulk carrier commercial ships. In tanker or LPG (liquefied petroleum gas) ships, more tanks are required for storing the loaded cargo (chemical liquids and others). The common types of tanks available on any commercial ship are seawater ballast tanks, fuel oil tanks and diesel oil tanks. In this article, the tank level measurement system on a bulk carrier ship will be discussed as a system based on using classical 4–20 mA pressure transmitters to perform the task of measuring the fluid levels in the ship’s tanks. A layout for the system was presented in [2]. Reviewing the maintenance and troubleshooting history of the system, it turned out that pressure transmitters dedicated to seawater ballast tanks were frequently replaced; however, those dedicated to fuel oil and diesel oil tanks were rarely replaced. Seawater ballast tanks are divided into two types, which are top side tanks and double bottom tanks. Each type of them is located in a different vertical level where top side tanks are located on a higher vertical level (main deck) than double bottom tanks (pipe tunnel). Therefore, the way of access for each type of these ballast tanks is determined by the restriction imposed by the ship’s structure. Transmitters dedicated to top side tanks are immersed through an upper flange into the tank, while those dedicated to double bottom tanks are horizontally mounted on an impulse line at the bottom of the tank. Temperature and moisture levels in the pipe tunnel area are relatively higher than those levels on the main deck; however, corrosion level on the main deck is relatively higher than that in the pipe tunnel area. These environmental conditions lead to the formation of ground loops, especially at those points where junction boxes are located to deliver 4–20 mA signals from pressure transmitters to input/output modules in the control system (Figure 1). Additionally, high salinity as well as a high level of impurities in seawater can be the main cause of shortening the transmitter’s lifetime due to their negative impact on the transmitter’s diaphragm as well as its cable, if it was immersed in the tank. Accordingly, and in order to improve the performance of the system, the following recommendations should be taken into account:

- Immersing pressure transmitters in seawater ballast tanks is not the best way to mount the transmitter (non-contact radar transmitters are a good option).
- Signal conditioners/isolators should be used at positions where junction boxes are located.
- Smart transmitters such as Foundation Fieldbus smart pressure transmitters can be a good alternative for classical 4–20 mA pressure transmitters with additional diagnostic information improving the reliability of the measurement process.

## 3. Smart Instrumentation Technologies (Theoretical Background)

### 3.1. HART Protocol (Hybrid Analogue–Digital Measurement and Control)

The HART protocol is a hybrid analogue–digital protocol in which additional digital information is superimposed on an analogue 4–20 mA current signal. The additional superimposed digital signal is an FSK (frequency shift keying) modulated signal in which ones are represented by a 1200 Hz signal while zeroes are represented by a 2200 Hz signal [2,3,4,5,6]. Basic principles of HART protocol were discussed in [2,3,4,5,6]. The average current of the superimposed digital signal is equal to zero. Therefore, the resultant current of HART signal (4–20 mA current + digital FSK superimposed signal) is equal to the current in the 4–20 mA analogue current loop. The superimposed digital signal includes diagnostic and parametric information for the field devices used in the measurement or control current loop. This additional information improves the reliability of the classical 4–20 mA current loop. HART protocol allows for point-to-point communication and multidrop communication modes [2,3,4,5,6]. In point-to-point communication, only a single field device can be connected to a single twisted pair of wires. In multidrop communication, a maximum number of 16 field devices can be connected to a single twisted pair of wires. Load resistance in an entire HART network should be between 230 ohms and 1100 ohms [2,3,4,5,6]. Load resistance of devices included in HART network can be calculated only at 20 mA load current. 

HART message structure consists of preamble, start character, address field, expansion field, command byte, byte count, status field, data field and checksum [5,7]. Each of these fields consists of a single byte or multiple bytes. Each byte is transmitted as an 11-bit UART character including a start bit, eight data bits, a parity bit and a stop bit. The HART message address field has two formats: short format and long format. Long format was firstly adopted by HART 5 version [5,7]. The hamming distance of the HART protocol is equal to four; therefore, it can detect up to three corrupted bits of data at one telegram. However, some higher-level communication errors may take place during transmission, and they will be detected by the status bytes at the field device side [5,7].

HART smart pressure transmitters such as Rosemount 3051S [8] provide more reliability to the 4–20 mA current loop. Reliability is provided by features such as baseline estimation and loop characterization. The baseline indicates the relation between the output current of the transmitter and the terminal voltage. Deviation of the baseline is a very important parameter based on which changes such as corrosion, water leak inside the transmitter or instability of power supply can be detected to maintain loop integrity. Such a property is turned off by default, but once the transmitter is installed, the user should start loop characterization. For proper characterization, an adequate amount of power is required. The transmitter will check the power level, and if it was within proper limits, it will generate an output current of 4 mA and 20 mA successively, and the correspondent terminal voltage for these current values will be recorded to estimate the baseline. A value should be assigned to the terminal voltage deviation limit parameter (default value is 1.5 V) through AMS (asset management system) device manager. If the terminal voltage deviation of the baseline will exceed this limit, an alert will be automatically generated. Loop characterization also provides an estimation of the loop resistance and loop power supply voltage, relying on the baseline calculations and comparison between previously and recently calculated baselines to detect changes in their values that might be caused by aging of the power supply or any physical changes in the loop condition [8].

### 3.2. Foundation Fieldbus (Wired Digital Measurement and Control)

The Foundation Fieldbus protocol is a digital communication protocol adopted by many smart transmitters. The basic principles of the Foundation Fieldbus protocol were briefly discussed in [2,9,10] ([11], pp. 21–55) [12,13]. Foundation Fieldbus IEC 61,158 is a fieldbus protocol that basically counts on the idea of using a single twisted pair of wires for the connection of multiple field devices, the role of which will be extended beyond the regular role of measuring process variables to the role of performing automation and control tasks independently of the authority of the master controller. Field devices perform such tasks by using the feature of distributed data transfer (DDT) functions [2,9,10] ([11], pp. 21–55) [12,13]. Foundation Fieldbus also has the ability of providing a reliable measurement and control operations in explosive hazardous application areas depending on intrinsically safe models such as the entity model, FISCO model, FNICO model, HPTC model and DART model. Foundation Fieldbus is very similar to Profibus PA; however, Profibus PA is more popular in Europe while Foundation Fieldbus is more popular in Asia and America. 

The Foundation Fieldbus signal is a Manchester-coded rectangular signal from the theoretical point of view; however, it is practically a trapezoidal waveform with rising and falling edges due to multiple factors such as delay imposed by modulation/demodulation electronic circuitry. The practical processing of a 31.250 Kbps Foundation Fieldbus Manchester-coded signal on the H1 bus was discussed in detail in [13], including modulation/demodulation techniques in noiseless as well as noisy conditions. The H1 bus is dedicated to the connection of all field devices along the same field bus; however, the HSE bus (high-speed ethernet) is dedicated to performing communication tasks between host controllers with a bit rate of 1–2.5 Mbps.

Foundation Fieldbus adopts a distributed communication system in which LAS (link active scheduler) plays an important role in controlling the communication [2,9,10] ([11], pp. 21–55) [12,13] process. For the purpose of redundancy, a single network may have two link masters, and in case one failed as the LAS, the other one will replace it. Communication between the LAS and field devices is divided into scheduled and unscheduled communication. 

Unscheduled communication is used for the transaction of diagnostic data and field device parameters. It takes place during breaks between scheduled communication intervals. Scheduled communication can be divided into two categories: the first one is related to control and measurement variables, while the second one is related to system management [2,9,10] ([11], pp. 21–55) [12,13]. In the first category, a field device periodically publishes its process data to the entire fieldbus buffer directly upon receiving a compel data command (CD) from the LAS [10,12,14]. In the second category, each field device will receive independent schedules (time distributions (TD)) for data transaction.

**Figure 2:**
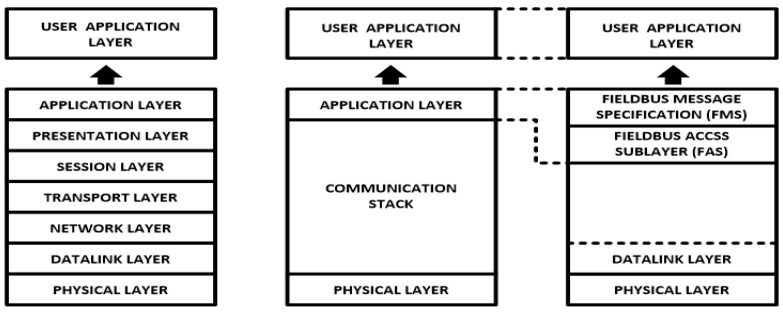
Foundation Fieldbus OSI (open systems interconnection) model.

As an OSI model (Figure 2), Foundation Fieldbus is divided into three layers, which are the user application layer, communication stack and physical layer [9,10] ([11], pp. 21–55) [12]. The communication stack performs only the roles of data link layer and application layer. A Foundation Fieldbus management system consists of two layers: the first one is the application layer, which is included in the communication stack, and the second one is the user application layer, which consists of function blocks and device description. The application layer included in the communication stack consists of fieldbus message specification (FMS) and a fieldbus access sublayer (FAS) [9] ([11], pp. 21–55). 

Field devices can be easily connected to the bus during operation through using the probe node (PN) command [10,12] issued by the LAS to detect the newly connected field devices to the bus. The field device will be automatically assigned an address directly after it responds to the PN command with the probe response (PR) command. Afterwards, the LAS cyclically issues a pass token (PT) command in order to check if the field device is still functional or not by acknowledging the device response to the transmitted PT. If the device fails to respond for several times, it will be automatically excluded from the field devices’ live list.

The statistical process monitoring block (SPM) [15] can be considered one of the most important function blocks at Foundation Fieldbus smart transmitters. Its task is to construct a noise signature of the transmitter primary variable with both mean and standard deviation values. SPM enables the transmitter to detect any sudden changes that may be related to some physical disturbances, such as propagation or vibration (swaying or pitching on a ship, for instance), which are not reflecting an actual real-time measured value. 

In a Rosemount 3051 FF smart pressure transmitter, the SPM block consists of three modules [15], which are:

- Statistical calculation module: The measured pressure values are applied to high-pass filter to detect any slow changes, such as set point modifications, and eliminate them while constructing the input signal noise signature. The mean value is calculated for the unfiltered signal, and the standard deviation will be calculated over the filtered signal.
- Learning module: responsible for establishing the process baseline values based on mean and standard deviation values calculated by the previous module.
- Decision module: it compares the measured value with the baseline, to decide if an alert/alarm should be activated or such a measured value should be ignored.

### 3.3. Wireless HART (Wireless Digital Measurement and Control)

Wireless HART is an extension of the HART protocol. The wireless HART specification (HART 7.1) was approved by the International Electrotechnical Commission (IEC) as a publicly available specification (IEC62591). In a wireless HART network, eight types of devices are involved. These devices are network managers, network security devices, access points, field devices, adapters, routers and handheld devices. All these devices form the mesh wireless HART network. Wireless HART has its own medium access control (MAC) sublayer in which the frame structure is called a superframe. The wireless HART physical layer supports the 2450 MHz ISM frequency band, which allows for up to 16 communication channels with a bandwidth of 2 MHz [16,17,18].

Wireless HART adopts time division multiple access (TDMA) [16,17,18] for the purpose of scheduling communication with field devices. Based on TDMA, communication tasks are performed during 10 ms time slots. A single time slot can be dedicated to communication with a single device or multiple devices. If a time slot is dedicated to communication with multiple field devices, it will be called a shared time slot. Synchronization between devices in a wireless HART network is required so that a successful TDMA can be maintained. Synchronization is maintained by using time synchronization mesh protocol (TSMP) [16,18,19]. In TSMP, transmission is accomplished when a single packet is transmitted, and an acknowledgement is generated that this packet was completely received without any errors. TSMP performs the role of transport layer, network layer and data link layer [16,18,19].

Direct sequence spread spectrum (DSSS) [16,17,18,20] is used to reduce overall signal interference by increasing the transmitted signal bandwidth. Only using DSSS will provide resistivity to the signal interference to a certain limit; however, using both the frequency-hopping spread spectrum (FHSS) [21] and DSSS leads to better interference rejection (FHSS) and higher coding gain (DSSS) [19]. FHSS depends on changing the frequency of the carrier signal with respect to time. The order of changing the carrier frequency should be known by both of the transmitter and the receiver [16,17,18,21].

In a wireless HART network, the data link sublayer is represented by the MAC protocol. The MAC protocol provides the mechanism that determines which user or device is allowed to access the medium when there is competition for it [16,17,18,22]. The MAC function [22] is called by the device when the device is about to transmit a message. The MAC function reads the device tables in order to check if the device is allowed to start transmission within the current time slot or not. The wireless HART network adopts TDMA for dedicated time slots while CSMA/CA (carrier sense multiple access with collision avoidance) [23,24] is adopted for shared time slots. CSMA/CA provides the mechanism of reducing the probability of collision between transmitted data using the exponential back-off algorithm [23,24].

Wireless HART network scheduling should be implemented on defined routes. Wireless HART adopts two ways of packet routing, which are source routing and graph routing [16,25]. In source routing, there is only one single route at the packet header to the destination device; however, for graph routing, charts are created for possible routes to the destination devices with a specific ID for each of these charts. The joint routing algorithm for maximizing network lifetime (JRMNL) [26] is another routing algorithm to improve the performance of a wireless HART network. It defines a link cost function to select the best next hop node to achieve the maximum network lifetime. The algorithm assigns degrees to nodes based on their hop distance to the gate (the degree assigned to the gate is zero). Then, the links between the nodes of the same degree are removed.

## 4. The 4–20 mA Analogue Standard (Integration and Performance Improvement)

The 4–20 mA analogue standard is the most popular analogue standard used for the purpose of measurement and control in maritime automation and instrumentation fields. The non-zero lower range limit of 4 mA in addition to its high level of immunity to noise sources are the main reasons behind the popularity of the 4–20 mA analogue standard. The main principles of the 4–20 mA analogue measurement and control current loop were discussed in [2]. As previously discussed, the tank level measurement system is based on the use of classical 4–20 mA pressure transmitters to measure the fluid levels in the ships’ tanks. 

**Figure 3:**
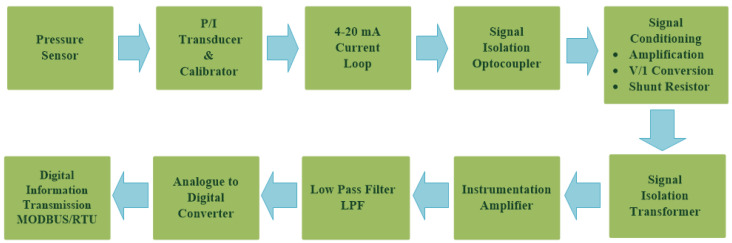
4–20 mA analogue pressure transmitter as a part of automation system.

**Figure 4:**
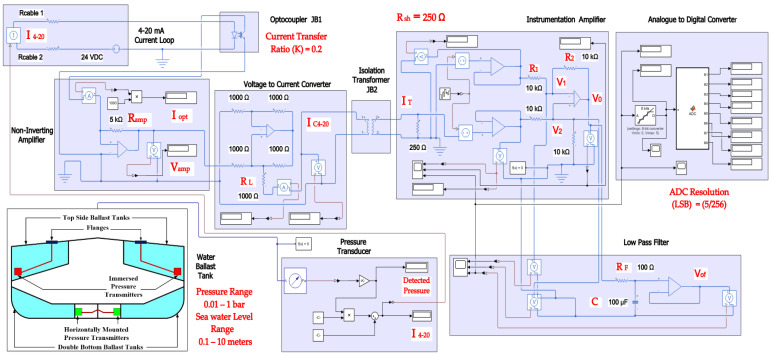
Simulink Simscape model used to simulate 4–20 mA pressure measurement current loop as a part of automation system.

**Figure 5:**
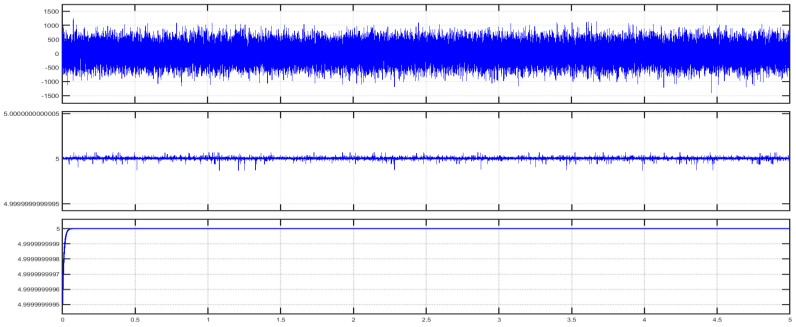
Illustration of 5 VDC voltage signal corresponding to 20 mA analogue current (1 bar of detected pressure). Upper figure illustrates voltage signal highly distorted by common mode noise at difference amplifier input. Middle figure illustrates the output voltage signal of instrumentation amplifier slightly distorted by coupled noise, while the lower figure illustrates output voltage after low-pass filtering.

In order to provide more comprehensive realization for the 4–20 mA analogue standard, it should be analyzed as a part of the whole instrumentation or automation system [27] (pp. 335–338). Figure 3 illustrates a block diagram for a Simulink Simscape model (Figure 4) that describes how a single 4–20 mA pressure measurement current loop is integrated into an automation system. Based on the connection diagram in Figure 1, each pressure transmitter consists of a pressure sensor and a P/I transducer. A pressure sensor detects the applied pressure to its diaphragm, while a pressure transducer converts the detected pressure into a 4–20 mA analogue current signal proportional to a supposed pressure detected from 0.01 to 1 bar (range calibration). The 4–20 mA controlled current source [2] simulates the generated analogue current proportional to the applied pressure. Cable resistance is taken into account [2] in the described model. The measured 4–20 mA (${\;I}_{4–20}$) analogue current is applied to an optocoupler to perform the first stage of signal isolation [27] (pp. 342–345), [28] recommended at junction boxes (J.B1). Assuming that the expanded optocoupler model in [29] was adopted to provide galvanic isolation at (J.B1), the output current of the optocoupler (${\;I}_{\mathrm{opt}}$) will be decreased linearly from the input current by a current transfer ratio (K) of 0.2 using Equation (1). Therefore, additional signal conditioning circuitry is needed to restore the original measured 4–20 mA signal. This circuitry includes a non-inverting amplifier and a grounded load voltage-to-current converter [30,31]. The output voltage of the non-inverting amplifier (${\;V}_{\mathrm{amp}}$) calculated by Equation (2) will be converted to the current (${\;I}_{C4–20}$) through the load resistance (${\;R}_{L}$) in order to restore the original 4–20 mA measured current from this amplified voltage signal through Equation (3). After the 4–20 mA measured signal was restored at the location of the first junction box near the transmitter, it will continue to flow through the two-wires cable to the junction box near the control system (J.B2) where additional signal isolation will be applied through an isolation transformer (the Simscape ideal transformer block can be used to represent either an AC transformer or a solid-state DC-to-DC converter, and the two electrical networks connected to the primary and secondary windings must each have their own electrical reference). The output current of the isolation transformer (${\;I}_{T}$) is applied to the analogue input module in the control system where a 250 ohms shunt resistor (${\;R}_{\mathrm{sh}}$) will be used to convert the 4–20 mA current signal into a 1–5 VDC voltage signal. The converted voltage signal will be applied to an instrumentation amplifier [32] to eliminate any common mode noise voltage signals, as calculated in Equation (4). The instrumentation amplifier includes two buffer amplifiers and one difference amplifier. The buffer amplifier is used for signal transfer from the high impedance side to the low impedance side of the op-Amp [32]. The difference amplifier detects the difference between the applied voltage to both the non-inverting input ($V_{1}$) and inverting input ($V_{2}$) of the op-Amp, which leads to elimination of any similar in phase signals such as common mode voltages (Figure 5) induced by common mode noise (represented by a source of additive white Gaussian noise applied to the non-inverting inputs of the two buffers) [32]. The output voltage signal of the instrumentation amplifier is applied to an active low-pass filter [33] to eliminate high-frequency coupled noise to the measured signal (Figure 5). The cut-off frequency ($f_{c}$) can be calculated using Equation (5). The analogue voltage output signal from the LPF ($V_{\mathrm{of}}$) is converted to a digital signal through using an 8-bit analogue-to-digital converter, which consists of a quantizer (quantized output is calculated through Equation (6) where *n* = 8 and $V_{\max}$ = 5 VDC) and MATLAB-based function (Appendix A) to convert the detected output of the quantizer into 8 bits of binary data. The binary output of the analogue-to-digital converter can be transmitted to the CPU of the system controller through serial communication interfaces (RS 232 or RS485) or serial communication protocols such as Modbus RTU (relatively popular in maritime applications) [2].

(1) $$K{=I}_{4–20}{/I}_{\mathrm{opt}}$$

(2) $$V_{\mathrm{amp}}{=I}_{\mathrm{opt}}{\;R}_{\mathrm{amp}}$$

(3) $$I_{C4–20}{=V}_{\mathrm{amp}}{/R}_{L}$$

(4) $$V_{o}{=(V}_{1}-V_{2})\;\frac{R_{2}}{R_{1}}$$

(5) $$f_{c}{=1/2\pi R}_{f}C$$

(6) $$\mathrm{Quantized}\;\mathrm{Output}=\mathrm{round}\left(\frac{2^{n}{\;V}_{\mathrm{of}}}{V_{\max}}\right)$$

## 5. Proposed Foundation Fieldbus (FF) Solution

As a smart alternative for classical 4–20 mA pressure transmitters, a more practical approach for Foundation Fieldbus will be discussed in this article. This practical approach relies basically on the replacement of classical 4–20 mA pressure transmitters used in the tank level measurement system on a bulk carrier commercial ship with Foundation Fieldbus pressure transmitters. Simulation models created by Emerson Segment Design Tool and Pepperl + Fuchs Segment Checker will simulate a safe area as well as explosive hazardous areas’ operational conditions. Therefore, the proposed solution will take into account possible non-intrinsically safe as well as intrinsically safe FF models, as follows:

- Non-intrinsically safe model.
- Intrinsically safe entity model.
- FNICO (Fieldbus Non-Incendive Concept) model.
- FISCO (Fieldbus Intrinsically Safe Concept) model.
- HPTC (High-Power Trunk Concept) model.
- DART (Dynamic Arc Recognition and Termination) model.

**Figure 6:**
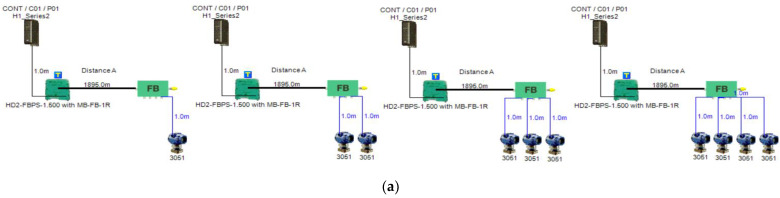

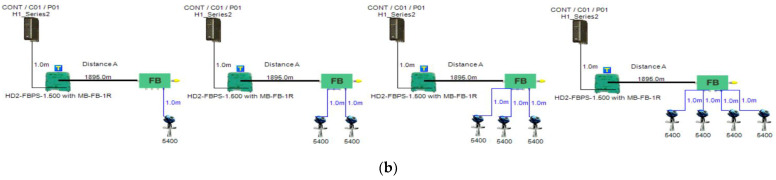
Test segments (**a**,**b**) dedicated to deriving the relation between main FF trunk cable length and current consumed by field barriers. (**a**) Test segments using Rosemount 3051 Pressure Transmitters; (**b**) Test segments using Rosemount 5400 Radar Transmitters.

**Figure 7:**
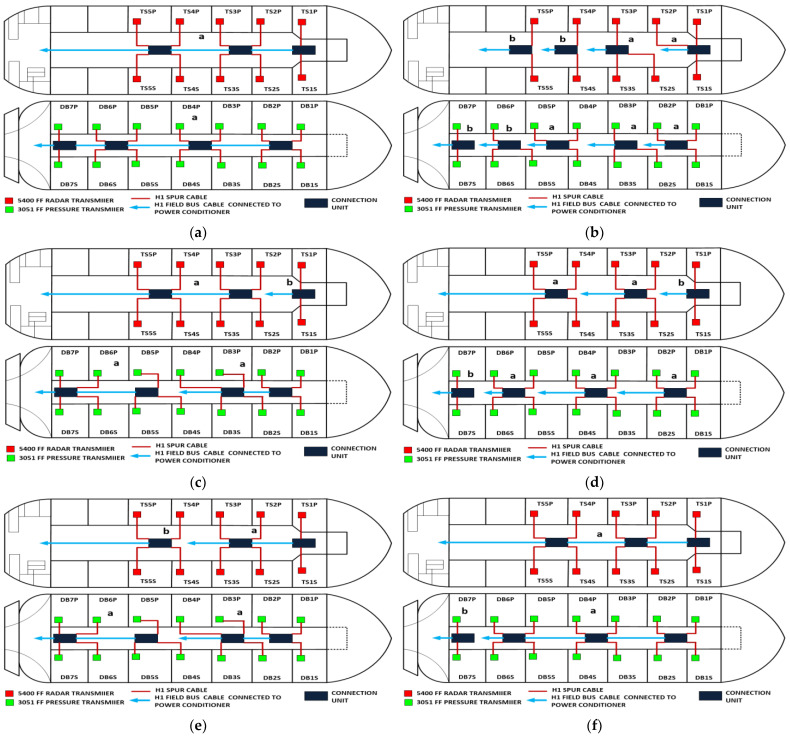

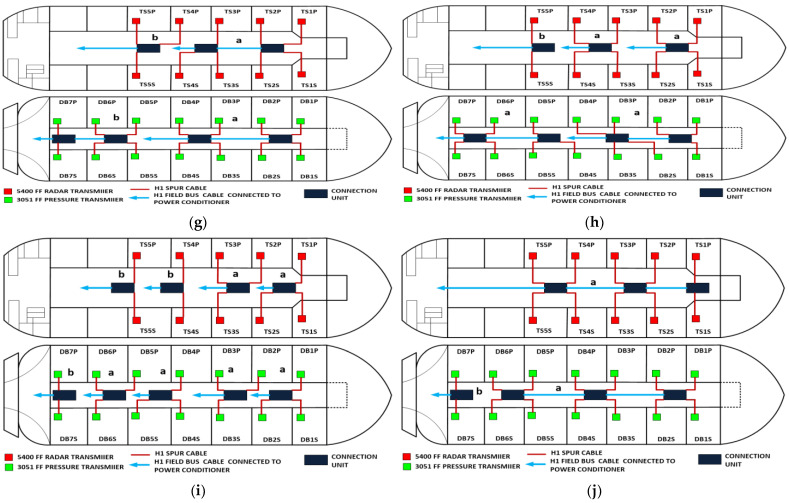
Layout of field device connection blocks for FF models. Connections units used differ according to the model (megablocks, megablocks with SC protection, field barriers or segment protectors). DB and TS refer to double bottom and top side tanks, respectively. S and P refer to starboard and port sides of the ship, respectively. Letters a and b on each layout refer to segments included in each sub-model as indicated in Table 1. (**a**) Non-intrinsically safe and HPTC model. (**b**) Intrinsically safe entity model. (**c**) Intrinsically safe FISCO IIB model. (**d**) Intrinsically safe FISCO IIC model. (**e**) Intrinsically safe FISCO IIB SC model. (**f**) Intrinsically safe FNICO IIB model. (**g**) Intrinsically safe FNICO IIB SC model. (**h**) Intrinsically safe FNICO IIC model. (**i**) Intrinsically safe FNICO IIC SC model. (**j**) Intrinsically safe DART model.

The proposed solution relies on replacement of the top side’s 10 immersed classical 4–20 mA transmitters with Foundation Fieldbus Rosemount 5400 non-contact radar transmitters and also replacement of the double bottom’s 14 classical 4–20 mA transmitters with Foundation Fieldbus Rosemount 3051 pressure transmitters. Each type of the previously mentioned models will be divided into two types of sub-models. The first one is for double bottom tanks, while the second one is for top side tanks. Each of these sub-models consists of a single or multiple segments according to the requirements imposed by each sub-model. The total number of segments is determined according to the maximum allowable current withdrawn from the segment power supply. The total current consumption in a FF segment $\left(I_{\mathrm{seg}}\right)$ is the sum of current consumed by the host ${\;(I}_{\mathrm{host}})$ and the current consumed by the H1 bus $\left(I_{H1}\right)$ as indicated in Equation (7). The segment power supply should be able to withstand the sum of both currents. The maximum capacity of the segment power supply is dependent of the FF model adopted by the segment. For the non-intrinsically safe model, entity model, FISCO, FNICO, DART and HPTC (only when segment protectors are used) models, the current consumed by the H1 bus is simply the sum of the current consumed by all field devices (transmitters and actuators) ${(I}_{d\;})$ if these devices were directly connected to the H1 bus without connection units such as megablocks; however, if those field devices were connected to the H1 bus through connection units, additional current will be consumed by these units. The current consumed by the connection unit is the sum of the operation current ${(I}_{c})$ and the current consumed by the field devices connected to the unit ${(I}_{d\;})$. In case the FF segment included connection units supporting short-circuit protection, Emerson Segment Design Tool calculates the short-circuit current $\left(I_{\mathrm{sc}}\right)$ only once (near the segment terminator) at the last connection unit with short-circuit protection connected to the bus. The reason the short-circuit current is calculated only once along the H1 bus is that all field devices are connected in parallel to the same field bus, so in case a short circuit occurred, the whole H1 bus will suffer a failure. For the HPTC model (when field barriers are used) and according to simulation results that will be discussed later, the current consumed by the field barriers is dependent on multiple additional factors as described in Section 5.5 in conjunction with test models illustrated in Figure 6. Distribution and location of connection blocks for each of the simulated models are illustrated in Figure 7, while Table 1 illustrates the connection diagram for each of the segments included in the sub-models.

(7) $$I_{\mathrm{seg}}{=I}_{\mathrm{host}}{+I}_{H1}$$

(8) $$I_{\mathrm{H1}}=\left\{\begin{array}{c} {\;I}_{d\;}\;\mathrm{Field}\;\mathrm{devices}\;\mathrm{are}\;\mathrm{connected}\;\mathrm{directly}\;\mathrm{to}\;\mathrm{the}\;H1\;\mathrm{bus}\\ {\;I}_{d}{+I}_{c}\;\mathrm{Field}\;\mathrm{devices}\;\mathrm{are}\;\mathrm{connected}\;\mathrm{to}\;\mathrm{the}\;H1\;\mathrm{bus}\;\;\mathrm{through}\;\mathrm{connection}\;\mathrm{units}\;\;\mathrm{without}\;\mathrm{short}\;\mathrm{circuit}\;\mathrm{protection}\\ {\;I}_{d}{+I}_{c}{+I}_{\mathrm{sc}}\;\mathrm{Field}\;\mathrm{devices}\;\mathrm{are}\;\mathrm{connected}\;\mathrm{to}\;\mathrm{the}\;H1\;\mathrm{bus}\;\mathrm{through}\;\mathrm{connection}\;\mathrm{units}\;\;\mathrm{with}\;\mathrm{short}\;\mathrm{circuit}\;\mathrm{protection} \end{array}\right.$$

Each FF segment should have at least two terminators. The power supply unit is provided with a built-in terminator, and the other terminator can be separately connected to the end of the bus, or the last connection unit might be provided with a built-in terminator similarly to the power supply, a Foundation Fieldbus terminator consists of a 100 Ω resistor connected in series with a 1µF capacitor. It is used as a current shunt for the control network, reducing the impact of reflections, noise and jitter. If more than two terminators are connected to the FF trunk, this will lead to high levels of distortion for the Manchester communication signal.

### 5.1. Non-Intrinsically Safe Model (Safe Area Application)

A non-intrinsically safe Foundation Fieldbus solution can be implemented with short-circuit protection or without short-circuit protection depending on the connection units used to connect field devices. The main features of the model can be explained as follows:

- Double bottom tanks’ sub-models: NON-IS-DB and NON-IS-DBSC.
- Top side tanks’ sub-models: NON-IS-TS and NON-IS-TSSC.
- Maximum capacity of power supply: 500 mA.
- $I_{c}$ for connection unit without short-circuit protection: 4 mA.
- $I_{c}{+I}_{\mathrm{sc}}$ for connection unit with short-circuit protection: (5 + 55) mA.
- Specifications of the sub-models and layout of connection units on the ship are indicated in Table 1 and Figure 7a, respectively.

### 5.2. Intrinsically Safe Entity Model

In the entity model, intrinsically safe calculations take into account the characteristics of the field bus cable (resistance, inductance and capacitance). Therefore, more restrictive ignition curves (inductive curves) will be adopted during ignition tests [11] (pp. 114–129) [34,35]. Consequently, the maximum available power through the field bus is decreased with respect to increased field bus cable length. The Foundation Fieldbus entity model allows for 2–3 field devices per segment depending on the available power provided by the power supply. An MTL5995 isolated power supply [36] will be used as a power supply conditioner, and an MTL5053 isolator [36] will be used as an intrinsically safe barrier. The main features of the model can be explained as follows:

- Double bottom tanks’ sub-model: IS-E-DB.
- Top side tanks’ sub-model: IS-E-TS.
- Maximum capacity of power supply: 80 mA.
- $I_{c}$ for connection unit without short-circuit protection: 0 mA.
- Specifications of the sub-models and layout of connection units on the ship are indicated in Table 1 and Figure 7b, respectively.

If short-circuit protection was used in the entity model, this will lead to segments with only one field device per segment because the current reserved for the connection unit with short-circuit protection will impose an additional load (55 + 5 mA) to the segment in which the available current provided by the power supply is only 80 mA, which leaves out almost 20 mA for the connection of field devices. For the Rosemount 3051 Pressure transmitter, which consumes almost 18 mA, it might be possible to connect the device; however, in such a case, the whole current consumed by the segment will be 78 mA, which is 97.5% of the available current by the power supply. Similarly, and since the available current for connecting the field device is 20 mA, it will not be possible to connect the Rosemount 5400 radar transmitter, the current of which is 21 mA. 

### 5.3. FISCO (Fieldbus Intrinsically Safe Concept) Model

The FISCO model is a Foundation Fieldbus intrinsically safe model that allows for a larger number of field devices per segment other than those allowed by the intrinsically safe entity model. The major difference between the entity model and FISCO model is that the FISCO model neglects cable reactance when performing intrinsically safe calculations. Based on experimental results, it was found that the value of cable reactance has no negative influence on ignition test results [37]. Therefore, Foundation Fieldbus trunk cable length has no effect on intrinsic safety conformation declaration. Accordingly, the maximum available power will not be affected by the bus cable length. FISCO allows for two types of power supplies according to the gas group adopted by the intrinsically safe FF network. For the IIB gas group (ethylene), FISCO power supplies allow for a maximum current of 265 mA with an output voltage of 13.1 VDC. For the IIC gas group (hydrogen), FISCO power supplies allow for a maximum current of 120 mA with an output voltage of 12.4 VDC ([11], pp. 114–129), [34,35,38]. The main features of the model can be explained as follows:

- Double bottom tanks’ sub-models: FISCO-IIB-DB, FISCO-IIC-DB and FISCO-IIB-DBSC.
- Top side tanks’ sub-models: FISCO-IIB-TS, FISCO-IIC-TS and FISCO-IIB-TSSC.
- Maximum capacity of IIB/IIC power supplies: 256 mA/120 mA.
- $I_{c}$ for connection unit without short-circuit protection: 0 mA.
- $I_{c}{+I}_{\mathrm{sc}}$ for connection unit with short-circuit protection: (5 + 55) mA.
- Specifications of the sub-models and layout of connection units on the ship are indicated in Table 1 and Figure 7c–e, respectively.

### 5.4. FNICO (Fieldbus Non-Incendive Concept) Model

The FNICO model is an intrinsically safe Foundation Fieldbus concept derived from the FISCO model. The major difference between the FNICO and FISCO models is that the FNICO model adopts a lower safety factor than the FISCO model in intrinsic safety calculations, that is why it is only applicable in Zone 2/Division 2 hazardous areas. Accordingly, it allows for higher values of available current and power than the FISCO model. FNICO allows for two types of power supplies according to the gas group adopted by the intrinsically safe FF network. For the IIB gas group (ethylene), FNICO power supplies allow for a maximum current of 320 mA with an output voltage of 13.1 VDC. For the IIC gas group (hydrogen), FNICO power supplies allow for a maximum current of 180 mA with an output voltage of 12.4 VDC [11] (pp. 114–129) [34,35,39,40]. The main features of the model can be explained as follows:

- Double bottom tanks’ sub-models: FNICO-IIB-DB, FNICO-IIC-DB, FNICO-IIB-DBSC and FNICO-IIC-DBSC.
- Top side tanks’ sub-models: FNICO-IIB-TS, FNICO-IIC-TS, FNICO-IIB-TSSC and FNICO-IIC-TSSC.
- Maximum capacity of IIB/IIC power supplies: 320 mA/180 mA.
- $I_{c}$ for connection unit without short-circuit protection: 0 mA.
- $I_{c}{+I}_{\mathrm{sc}}$ for connection unit with short-circuit protection: (5 + 55) mA.
- Specifications of the sub-models and layout of connection units on the ship are indicated in Table 1 and Figure 7f–i, respectively.

### 5.5. HPTC (High-Power Trunk Concept) Model

Unlike FISCO and FNICO models, the High-Power Trunk Concept does not impose any limitations on the maximum available power at the Fieldbus trunk cable, which allows for longer cable lengths and a higher number of field devices per segment. The High-Power Trunk Concept allows for an output voltage up to 30 VDC and a maximum current up to 500 mA. The basic idea of a High-Power Trunk Concept is to provide unlimited energy to the field bus trunk; however, within the hazardous area, this unlimited energy will be distributed using energy-limiting wiring interfaces till it is delivered to the field device. HPTC does not require power supply conditioners particularly dedicated to the model, as standard non-intrinsically safe lower price power supplies can be used in the HPTC network. Energy-limiting wiring interfaces include field barriers and segment protectors. They both provide short-circuit protection as well as galvanically isolated outputs [11] (pp. 114–129) [34,35,41]. Each of these outputs acts as a FISCO or entity power supply. HPTC allows for a maximum number of four field barriers. Each of these barriers allows for up to four field devices. Therefore, HPTC allows for up to 16 field devices per segment. According to the simulation results that will be discussed later, there are two major differences between segment protectors and field barriers. The first difference is related to the available voltage at the output terminals to which field devices are connected. Segment protectors maintain constant voltage at a specific field device connected to a specific segment protector regardless of the voltage drop on the spur. Field barriers maintain constant output voltage all along the segment at the output port dedicated to a specific field device regardless of the total voltage drop on the H1 bus main trunk cable. Segment protectors are used with Zone2/Div2 applications; however, field barriers are used with Zone1/Div2 applications. Therefore, the voltage available at field barrier output terminals connected for field devices is less than the voltage available at segment protector output terminals for field devices, as will be shown in the simulation results. The second difference between segment protectors and field barriers, which was observed during simulation, is that the value of the current consumed by each field barrier $I_{\mathrm{FB}}$ is dependent of the following variants:

1. The total current of field devices connected to the field barrier ($I_{\mathrm{dT}}$).
2. The H1 bus main trunk overall cable length (${\;L}_{T}$).
3. Number of field barriers included in the segment ($N_{\mathrm{FB}}$) and current consumed by each of them (${\;I}_{\mathrm{dTS}}$).
4. Length of H1 bus main trunk cable sections between field barriers (${\;L}_{\mathrm{FB}}$).
  (9) $$I_{\mathrm{FB}}{=f\;(\;I}_{\mathrm{dT}}{,\;I}_{\mathrm{dTS}}{,\;N}_{\mathrm{FB}}{,\;L}_{\mathrm{FB}}{,\;L}_{T})$$

The HPTC model is divided into two sections: the first section is based on using segment protectors, while the second section is based on using field barriers. The main features of the model can be explained as follows:

- Double bottom tanks’ sub-models: HPTC-SP-DB and HPTC-FB-DB.
- Top side tanks’ sub-models: HPTC-SP-TS and HPTC-FB-TS.
- Maximum capacity of power supply: 500 mA.
- $I_{c}{+I}_{\mathrm{sc}}$ for segment protectors: (4 + 58) mA.
- Current consumed by field barriers is dependant of the previously mentioned variants, as explained in Equation (9).
- Specifications of the sub-models and layout of connection units on the ship are indicated in Table 1 and Figure 7a, respectively.

In order to define the characteristics of the relation between some of the previously mentioned variants and the current consumed by the field barrier, simple HPTC segments (Figure 6) were constructed using Emerson Segment Design Tool to derive the relation between the first two variants ($I_{\mathrm{dT}}$ and $L_{T}$) and the current consumed by the field barrier. Four of these segments are dedicated to Rosemount 3051 transmitters (Figure 6a), while the other four segments are dedicated to Rosemount 5400 transmitters (Figure 6b). In each of these segments, the current consumed by field barriers will be calculated with respect to the change in the distance A between the field barrier and the power supply from 10 m to 1895 m, with increments of 10 m. These calculations will be performed when one, two, three or four transmitters are connected to the field barrier.

### 5.6. DART (Dynamic Arc Recognition and Termination) Model

The DART intrinsically safe model is the latest intrinsically safe fieldbus segment design system. The basic idea of any intrinsically safe system is the continuous limitation of the energy in hazardous areas below a specific level that may lead to ignition. This energy level might be reached either by sparking or by heating. DART adopts an alternative technique to prevent ignition. This technique is based on detecting the spark current rate of change with respect to time. Based on the detected spark current rate of change, DART will limit the H1 bus energy only during the first 5–10 microseconds of spark formation, which leads to extinguishing the spark before it will become incendive. Emerson Segment Design Tool does not provide the possibility of simulation for the DART model and that is why the Pepperl + Fuchs Segment Checker simulation program was used to simulate the proposed DART model. Unlike the HPTC model, which allows for a regular segment power supply, the DART model allows only for dedicated power supplies with a maximum current of 360 mA. The proposed DART model consists of two sub-models (DART-DB and DART-TS). Specifications of the sub-models and layout of connection units on the ship are indicated in Table 1 and Figure 7j, respectively [34,35,42,43]. 

## 6. Results

**Figure 8:**
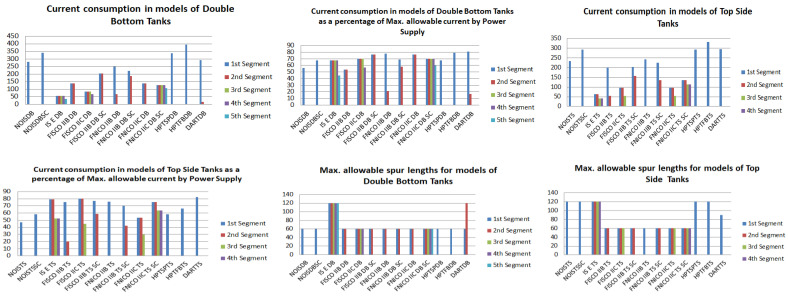
Total current consumption and maximum allowable spur lengths of all sub-models.

**Figure 9:**
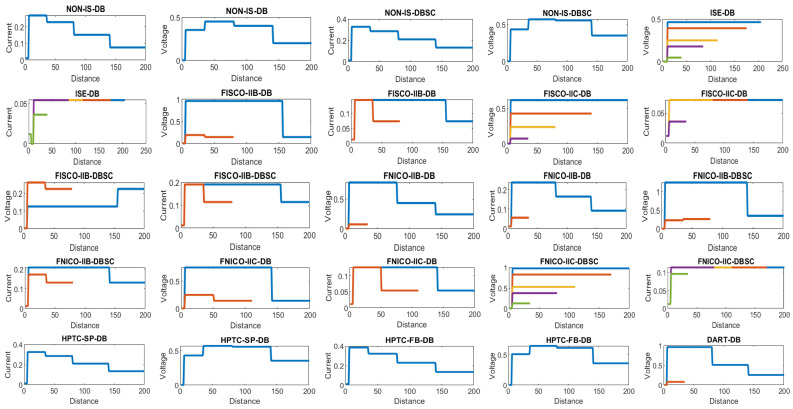

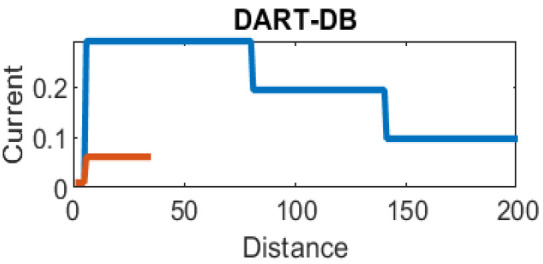
Current and voltage drop on the cables included in sub-models dedicated to double bottom tanks. Blue, brown, yellow, violet and green colors refer to segments from 1 to 5, respectively.

**Figure 10:**
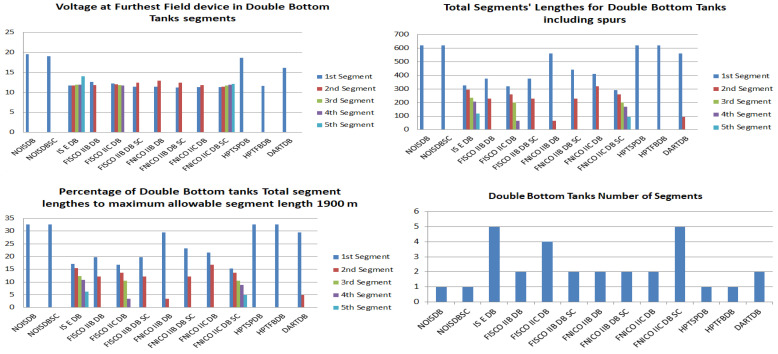
Illustration of voltage at farthest field devices, total segments’ lengths and percentage of them to the maximum allowable length of 1900 m and total number of segments for double bottom tanks’ sub-models.

**Figure 11:**
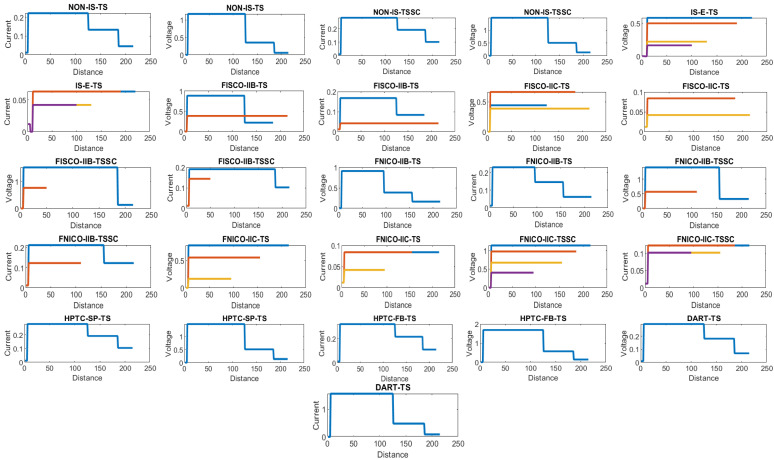
Current and voltage drop on the cables included in sub-models dedicated to top side tanks. Blue, brown, yellow and violet colors refer to segments from 1 to 4, respectively.

**Figure 12:**
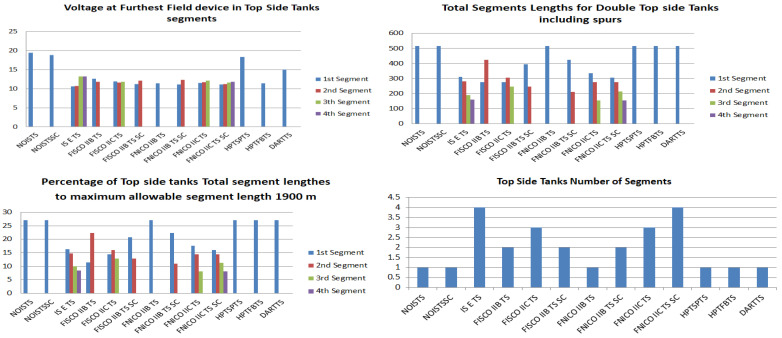
Illustration of voltage at farthest field devices, total segments’ lengths and percentage of them to the maximum allowable length of 1900 m and total number of segments for top side tanks’ sub-models.

Figure 8 illustrates the total current consumption, percentage of total current consumption to the maximum allowable current by power supply and maximum allowable spur length for each of the segments in the simulated sub-models. Voltage drops on the H1 bus cable sections are calculated for all the sub-models in Table 1. Figure 9 illustrates current and voltage drops on the H1 bus cable sections included in the sub-models dedicated to double bottom tanks. Figure 10 illustrates the total number of segments, voltage at farthest field devices, total segments’ lengths and percentage of these lengths to the maximum allowable length of 1900 m for models dedicated to double bottom tanks. Figure 11 and Figure 12 illustrate the same parameters for models dedicated to top side tanks similarly to Figure 9 and Figure 10 respectively.

**Figure 13:**
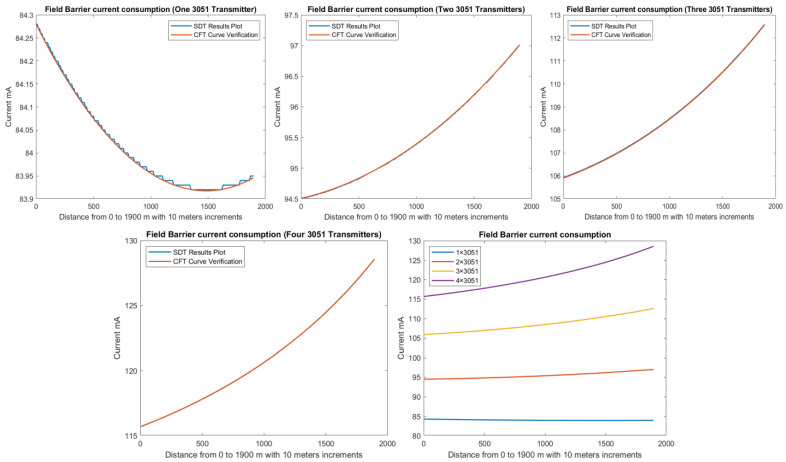
Illustration of current consumed by field barrier with respect to FF H1 trunk cable length when for four models in which one, two, three or four 3051 transmitters were connected to the field barriers in the models in Figure 6a.

**Figure 14:**
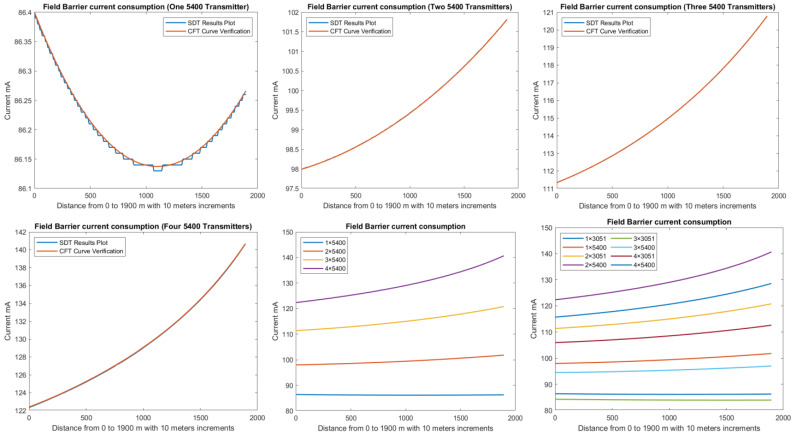
Illustration of current consumed by field barrier with respect to FF H1 trunk cable length when for four models in which one, two, three or four 5400 transmitters were connected to the field barriers in the models in Figure 6b. (Last plot for both 3051 and 5400 transmitters.)

Figure 6 illustrated eight models dedicated to deriving the relation between the current consumed by field barriers and current consumed by field devices connected to the field barrier $I_{\mathrm{dT}}$ as well as H1 bus cable length ${\;L}_{T}$ between the field barrier and segment power supply for both the Rosemount 3051 FF pressure transmitter and Rosemount 5400 radar transmitters. Results of these simulation models were plotted using MATLAB. Curve Fitting Tool in MATLAB was used to derive the mathematical equations describing the obtained simulation plots for each of the models in Figure 6. The mathematical equations obtained were verified by the same MATLAB code providing curves similar or identical to the simulation plots. These mathematical equations characterized the relation between field barriers’ current consumption and length of H1 bus cable (for a specific number of field devices connected to the barrier) as a polynomial relation of the fourth degree. This polynomial relation is generally described in Equation (10) where $I_{\mathrm{FB}}$ is the current consumed by the field barrier, and $L_{T}$ is the length of the H1 bus cable between the power supply and field barrier. The values of coefficients $k_{1}$, $k_{2}$, $k_{3}$, $k_{4}$ and $k_{5}$ are used to distinguish between the eight models illustrated in Figure 6a,b. The values of these coefficients for each of these eight models are indicated in Table 2. Figure 13 and Figure 14 both illustrate the SDT (Segment Design Tool) simulation plots as well as the equation verifying curves for the models illustrated in Figure 6a,b, respectively. It would be worth mentioning that the obtained equations are applicable only for the models illustrated in Figure 6, which means that for another segment structure, where more field barriers as well as more field devices are added, the current consumed by the field barriers will be dependent of not only $I_{\mathrm{dT}}$ and $L_{T}$ (similarly to segments in Figure 6) but also dependent of ${\;I}_{\mathrm{dTS}}{,\;N}_{\mathrm{FB}}$ and $L_{\mathrm{FB}}$.

(10) $$I_{\mathrm{FB}}{=k}_{1}{\;L}_{T}{}^{4}{+k}_{2}{\;L}_{T}{}^{3}{+k}_{3}{\;L}_{T}{}^{2}{+k}_{4}{\;L}_{T}{+k}_{5}$$

## 7. Discussion

### 7.1. The 4–20 mA Pressure Measurement Current Loop on a Bulk Carrier Commercial Ship

In order to ensure an increased reliability for the measurement process in the tank level measurement system based on using classical 4–20 mA analogue transmitters on a bulk carrier ship, this article presented a Simulink Simscape model that simulated a pressure measurement 4–20 mA current loop in the tank level measurement system on a bulk carrier commercial ship as an integrated part of the automation system. Conditions which might impair the reliability of the measurement process were taken into account during simulation. Impairment rendered by such conditions (related to the specific nature of the maritime environment, such as high levels of temperature, moisture, corrosion and salinity) can be manifested in the formation of ground loops or induction of coupled noise as well as common mode noise. The model proposed using signal isolators such as optocouplers or transformer isolators to cut the galvanic path between different possible unwanted ground points originated from previously mentioned environmental effects in specific locations along the pressure measurement current loop. Junction boxes connecting between the transmitter and the control system on the main deck or in the pipe tunnel area are examples for such locations where ground loops are most likely to occur. On the other hand, the model simulated elimination of common mode voltages induced by common mode noise through using instrumentation amplifiers, which are usually located inside analogue input cards in the control system. Additionally, the Simulink Simscape model also illustrated how to eliminate coupled noise along the pressure measurement 4–20 mA current loop through using active low-pass filters to eliminate higher-frequency unwanted signals. Functional safety is also a means by which performance of the 4–20 mA measurement current loop can be improved. In a functionally safe 4–20 mA current loop, redundancy is implemented by duplicating each of the components included in the loop, especially signal isolators/conditioners and instrumentation amplifiers. Detailed research dedicated to the implementation of a functionally safe 4–20 mA current loop for maritime application will be conducted in the near future.

### 7.2. Foundation Fieldbus Solution (Comparative Analysis) 

This article provided a detailed simulation of using Foundation Fieldbus transmitters as an alternative for classical analogue 4–20 mA transmitters in the tank level measurement system on a bulk carrier ship. Radar non-contact transmitters were chosen particularly to replace 4–20 mA classical immersed pressure transmitters, as was recommended in [2] in order to avoid possible negative effects induced by immersing the transmitter in seawater with high levels of salinity and impurities, which usually leads to shortening the transmitter’s lifetime. Both Rosemount FF transmitters (3051 and 5400) were chosen for such an analysis, as both of them are intrinsically safe-certified transmitters compatible for applications in explosive hazardous areas. Twenty-six non-intrinsically safe and intrinsically safe sub-models for both double bottom and top side tanks were discussed in detail (Table 1). The intrinsically safe sub-models were based on entity, FISCO, FNICO, HPTC and DART concepts. These sub-models were comparatively analyzed to identify the major differences between them not only from a theoretical point of view but also from a perspective related to the conducted simulation and MATLAB-based analysis for the results obtained from such a simulation. On the other hand, and since control and measurement systems on commercial ships are quite similar, simulation conducted for possible intrinsically safe FF models for the tank level measurement system on a bulk carrier ship can be applied for the same system on other types of commercial ships where higher safety considerations are required, such as chemical tanker ships and LPG ships. The analysis of simulation results revealed many points of interest based on which future experimental research can be conducted to verify the authenticity of such results, as well as the degree of similarity between simulation models and real-time applications. These points of interest can be described as follows:

- Highest number of segments required to implement an FF model for DB and TS tanks was observed at entity model. Among FF proposed models, entity model allows for minimum available power to the trunk, a consequently minimum number of allowable field devices and a consequently higher number of segments for the same model.
- Applying short-circuit protection reduces the maximum number of allowable field devices per segment, as the power supply should be able to withstand the short-circuit current in case it takes place. Short-circuit current is calculated only once for the last connection unit at the field bus.
- Unlike all intrinsically safe FF models, the HPTC intrinsically safe model is the only model that allows for segment power supply units that are not particularly dedicated to HPTC, and that is why non-intrinsically safe models are almost identical to HPTC models except for the difference that segment protectors or field barriers are used in HPTC models as connection units for field devices, while simple connection megablocks are used in non-intrinsically safe models.
- In entity intrinsically safe models, there is no current flow in the cable connecting between the MTL5995 power supply and MTL5053 intrinsically safe barrier, and accordingly, the voltage drop on the same cable will be 0 VDC.
- For all FF models except for the HPTC model, voltage drop at any field device in the segment can be calculated by Equation (11) where $V_{\mathrm{supply}}$ is the available voltage from the segment power supply, $V_{H1}$ is the voltage drop on the H1 bus main trunk cable from the power supply to the device, $V_{c}$ is the voltage drop on the connection unit to which the field device spur is connected and $V_{\mathrm{spur}}$ is the voltage drop on the spur to which the field device is connected. For all connection units (megablocks, segment protectors and field barriers), there is no voltage difference between input and output terminals connected to the H1 bus main trunk cable. $V_{H1}$ is dependent of the total current consumption in the segment and overall length of the main H1 bus trunk cable, while $V_{\mathrm{spur}}$ is dependent of spur length and field device current connected to the spur. The voltage drop on any connection unit $V_{c}$ exists between the input terminals connected to the H1 bus main trunk cable and the output terminals dedicated to spur connections. In the simulated models, $V_{c}$ was equal to zero in all models except for the HPTC model based on using field barriers and segment protectors. In the DART model, $V_{\mathrm{supply}}$ will be decreased only in the first 5–10 microseconds of possible spark ignition in order to reduce the voltage delivered to the field device $V_{d}\;$ so that the spark would be early extinguished.
  (11) $$V_{d}{=V}_{\mathrm{supply}}{\;-\;V}_{H1}{\;-\;V}_{c}{-\;V}_{\mathrm{spur}}$$
- In HPTC sub-models with the segment protectors HPTC-SP-TS and HPTC-SP-DB, constant voltage was maintained at field devices, irrespective of the voltage drop on the spur. When increasing the spur length to which the field device is connected, the segment protector will increase the voltage at the output terminals to compensate the increased voltage drop on the spur. The output voltage of the segment protector is decreased from the segment protector input voltage by a reduction coefficient, the value of which for a specific field device will decrease when increasing the spur length. In HPTC sub-models with the field barriers HPTC-FB-TS and HPTC-FB-DB, output ports dedicated to connecting field devices of a specific current maintained constant voltage all along the segment, irrespective of the voltage drop on the H1 bus trunk main cable. This constant output voltage is maintained through compensating the decreased input voltage at farther field barriers by a reduction coefficient, which is decreasing from the nearest field barrier to the farthest field barrier in the segment. For an HPTC segment consisting of K connection units (field barriers or segment protectors), each (k) connection unit has N output ports for connecting field devices. For a field device (n) connected to the connection unit (k), the voltage level at the field device ($V_{\mathrm{dHPkn}}$) will be the difference between output voltage form the connection unit at the port connected to the device ($V_{\mathrm{oHPkn}}$) and the voltage drop on the spur to which the device is connected ($V_{\mathrm{sHPkn}}$). The input voltage to the (k) connection unit ($V_{\mathrm{iHPk}}$) is reduced by a reduction coefficient ($K_{\mathrm{vHPkn}}$) the value of which is dependent on the type of the connection unit, whether it was a field barrier or a segment protector. If the connection unit was a segment protector, the value of the reduction coefficient will be a function of the field device current ($I_{\mathrm{dHPkn}}$) and voltage drop on the spur to which the field device is connected. If the connection unit was a field barrier, the value of the reduction coefficient will be a function of the field device current and the sum of the voltage drops on the H1 bus main trunk cable from the power supply to the field barrier. Accordingly, and due to equal spur lengths and equal field devices’ currents in HPTC sub-models, the voltage available at all field devices was a constant value (11.626 VDC and 11.397 VDC for HPT-FB-DB and HPT-FB-TS, respectively).
  (12) $$V_{\mathrm{dHPkn}}{=V}_{\mathrm{oHPkn}}{\;-\;V}_{\mathrm{sHPkn}}$$
  (13) $$V_{\mathrm{oHPkn}}{=V}_{\mathrm{iHPk}}{\;-\;K}_{\mathrm{vHPkn}}$$
  (14) $$V_{\mathrm{iHPk}}{=V}_{\mathrm{supply}}-\;\overset{k}{\underset{j=1}{\sum }}V_{H1j}\;k\;\in \;\{1,2,\ldots ,K\}\;\mathrm{and}\;n\;\in \;\{1,2,\ldots ,N\}$$
  (15) $$K_{\mathrm{vHPkn}}=\left\{\begin{array}{c} {\;f\;(\;I}_{\mathrm{dHPkn}}{\;,\;V}_{\mathrm{sHPkn}}\;)\;For\;Segment\;Protectors\;\\ {\;f\;(\;I}_{\mathrm{dHPkn}}\;,\;\overset{k}{\underset{j=1}{\sum }}V_{H1j}\;)\;For\;Field\;Barriers \end{array}\right.$$
- Voltage level at farthest field device in the HPTC segment where field barriers are used is less than the voltage level at farthest field devices where segment protectors are used for a similar segment due to the fact that field barriers are dedicated to Zone1/Div2 applications, while segment protectors are dedicated to Zone2/Div2 applications.
- Current consumed by field barriers in HPTC models is a function of $I_{\mathrm{dT}}{,\;I}_{\mathrm{dTS}}{,\;N}_{\mathrm{FB}}{,\;L}_{\mathrm{FB}}$ and $L_{T}$. Simple HPTC test models were constructed to simulate the effect of gradual increase of the overall H1 bus cable length $L_{T}$ (10–1895 m with increments of 10 m) on the current consumed by field barriers $I_{\mathrm{FB}}$ for different values of $I_{\mathrm{dT}}$. Analysis of simulation results showed that the relation between $I_{\mathrm{FB}}$ and $L_{T}$ is a polynomial relation identified by a group of coefficients dependent on different values for the sum of the current consumed by field devices connected to the field barrier $I_{\mathrm{dT}}$. Due to this polynomial relation, $I_{\mathrm{FB}}$ will increase with increasing $L_{T}$. The increasing rate of $I_{\mathrm{FB}}$ with respect to $L_{T}$ is proportional to values of $I_{\mathrm{dT}}$. For lower values of $I_{\mathrm{dT}}$ (when only a single transmitter is connected to the barrier), an initial decrease in $I_{\mathrm{FB}}$ was noticed with respect to increasing $L_{T}$, and then $I_{\mathrm{FB}}$ will continue to follow the polynomial curve increasing with respect to increasing $L_{T}$. The lower was the value of $I_{\mathrm{dT}}$, the higher will be the range of the H1 bus trunk cable length $L_{T}$ through which $I_{\mathrm{FB}}$ will be decreasing. This range was almost 1490 m when a single Rosemount 3051 pressure transmitter ($I_{\mathrm{dT}}$ = 18 mA) was connected to the field barrier, while it was almost 1100 m when a single Rosemount 5400 pressure transmitter ($I_{\mathrm{dT}}$ = 21 mA) was connected to the field barrier. In these test models where only a single field device was connected to the field barrier, it was noticed that Emerson Segment Design Tool follows a discrete curve other than the continuous curves adopted when two, three or four transmitters were connected to the field barrier. Further detailed experimental research will be conducted in order to verify the degree of similarity between simulation and experimental results. Similarly, and for the purpose of identifying the relation between $I_{\mathrm{FB}}$ and rest of the variables $I_{\mathrm{dTS}}{,\;N}_{\mathrm{FB}}$ and $L_{\mathrm{FB}}$, future research will be conducted.
- The non-linear behavior of both of current consumption and output voltage in HPTC connection units (segment protectors and field barriers) is a clear demonstration of the technique adopted by the HPTC model to distribute the overall energy consumed in the segment on each of the connection units included in the segment. This demonstration is particularly depicted in the dependence of the current consumed by a specific field barrier on the current consumed by other field barriers in the segment, whether they preceded or followed that specific field barrier. Moreover, the current consumed by that specific field barrier is also dependent on the lengths of the H1 bus main trunk cable sections before the field barrier as well as after the field barrier.
- According to the simulation results obtained from Emerson Segment Design Tool, Table 3 illustrates the maximum allowable spur lengths in different Foundation Fieldbus models. For non-intrinsically safe, HPTC and DART models, maximum allowable spur length is dependent on the total number of field devices per segment; however, for entity, FISCO and FNICO models, the maximum allowable spur length in the segment is a constant value independent of the total number of field devices per segment.
- As illustrated in Figure 10 and Figure 12, current consumption and voltage drops at H1 bus cable sections in the sub-models HPTC-FB-DB and HPTC-FB-TS are higher than current consumptions and voltage drops at H1 bus cable sections in similar sub-models NONIS-DB and NONIS-TS, respectively (sharing the same layout, dimensions and number of segments). This can be explained due to the non-linear (polynomial) characteristics of current consumed by field barriers in the HPTC model with respect to bus cable length. The current flowing in the H1 bus trunk cable where field barriers are used will be more than the current flowing in H1 bus trunk cable where regular connection units are used. Consequently, this results in higher voltage drops on H1 bus cable sections in comparison with all other FF models for similar segments in which the value of the current flowing in the H1 bus cable is independent of the H1 bus cable length and only dependent on total number of field devices connected to the bus and their currents. Similarly, and due to the same explanation, the highest total current consumption for a segment was observed at the HPTC model, as illustrated in Figure 9.

## 8. Conclusions

- In order to improve the performance of the 4–20 mA analogue standard in maritime engineering applications, signal isolators/conditioners are recommended to be installed at junction boxes located at areas subjected to higher levels of harsh environmental conditions such as the main deck and pipe tunnel so that ground loops can be avoided.
- Instrumentation amplifiers and low-pass filters are used to eliminate common mode noise voltages and coupled noise, respectively. They can be integrated inside analogue input/output modules or used separately.
- Using smart transmitters based on wired (HART and Foundation Fieldbus) or wireless (wireless HART) communication protocols can be an alternative for classical 4–20 mA analogue transmitters in maritime engineering applications, increasing reliability as well as stability of the measurement/control process with additional diagnostic and parametric information. This article considered the possible use of FF models in safe as well as explosive hazardous areas.
- For FF intrinsically safe models, the entity model requires the highest number of segments.
- Using FF connection units with short-circuit protection imposes an additional current that the segment power supply should be able to withstand in case of short-circuit occurrence.
- Short-circuit current in FF segments with short-circuit protection units is calculated only once for the last connection unit in the segment.
- There is neither current flow nor voltage drop at the cable section connecting between the segment power supply and intrinsically safe barrier in the FF entity model.
- The HPTC intrinsically safe model is the only FF model that does not require dedicated segment power supplies for it.
- Current consumed by field barriers in the HPTC intrinsically safe model is non-linearly (polynomial relation) related to H1 bus overall cable length and lengths of the H1 bus cable sections between the field barriers and total number of field devices connected to all field barriers in the segment.
- In the HPTC model based on field barriers, the voltage level at each of the output ports dedicated to connecting a specific field device is a constant voltage reduced from the input voltage to the field barrier by a reduction coefficient, the value of which is dependent on the field device current and the voltage drop on the H1 bus main trunk cable. The value of the reduction coefficient is decreased when the overall H1 bus cable length is increased.
- In the HPTC model based on segment protectors, the voltage level at a specific field device connected to a specific segment protector is a constant voltage less than the input voltage to the field barrier by a reduction coefficient, the value of which is dependent of the field device current and the voltage drop on the spur cable to which the device is connected. The value of the reduction coefficient is decreased when spur length connected to the field device is increased.
- Maximum allowable spur length in the segment is dependent on the number of field devices in the segment for non-intrinsically safe, HPTC and DART models.
- Maximum allowable spur length in the segment is independent of the number of field devices in the segment for entity, FISCO and FNICO models.
- In a single FF segment, the highest current consumption in H1 bus cable sections is at the closest section to the segment power supply connecting between the power supply and first connection unit; however, for the voltage drop on H1 bus cable sections, it is not a condition that sections with the highest current should have the highest voltage drop across them, as it is also dependent on the length of cable section.
- Higher FF segment lengths are observed with models that consist of a lower number of segments.
- For similar segments (sharing the same layout and connection diagram), current flow in the H1 bus cable as well as voltage drop on the same H1 bus cable, where the HPTC model (with field barriers) is adopted, is higher than the current flow and voltage drop in the H1 bus cable of the same length where other FF models will be adopted.

## Figures and Tables

**Table 1:** Connection layout for each of the proposed Foundation Fieldbus models. Type of cables used in all models is FF type A 0.8 ${\mathrm{mm}}^{2}$ (18 AWG). S/M (Sub-Model), No.S (No. of segments), No.T/S (No. of Transmitters/Segments), P/S (Power Supply), A/V (Available Voltage), C/U (Connection Unit), T.Cur (Total Current Consumption), R.Cur (Rated Current), Max.S/L (Maximum Spur Length).

FF Sub-Model	Layout of Field Device Connection Blocks
S/M: NONIS-DB Figure 7a No.S: a(1) No.T/S: a(14) P/S: HD2-FBPS 1 2.3.500 A/V: 21 VDC C/U: FCS-MB4 T.Cur: 280 mA T.Cur/R.Cur = 56% Max.S/L: length: 60 m	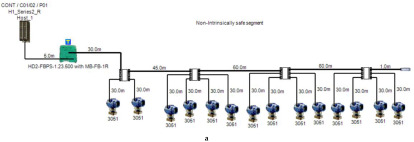
S/M: NONIS-DBSC Figure 7a No.S: a(1) No.T/S: a(14) P/S: HD2-FBPS 1 2.3.500 A/V: 21 VDC C/U: JBBS-49SC-E413 T.Cur: 339 mA T.Cur/R.Cur = 67.8% Max.S/L: 60 m	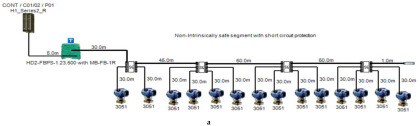
S/M NONIS-TS Figure 7a No.S: a(1) No.T/S: a(10) P/S: HD2-FBPS 1 2.3.500 A/V: 21 VDC C/U: FCS-MB4 T.Cur: 234 mA T.Cur/R.Cur = 46.8% Max.S/L: 120 m	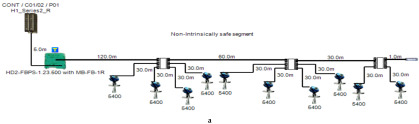
S/M: NONIS-TSSC Figure 7a No.S: a(1) No.T/S: a(10) P/S: HD2-FBPS 1 2.3.500 A/V: 21 VDC C/U: JBBS-49SC-E413 T.Cur: 292 mA T.Cur/R.Cur = 58.4% Max.S/L: 120 m	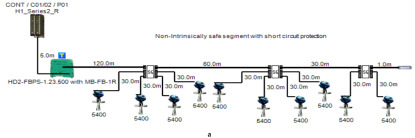
S/M: IS-E-DB Figure 7b No.S: a(4), b(1) No.T/S: a(3), b(2) P/S: MTL5995- MTL5053 A/V: 12.168 VDC C/U: 6 Port Brick T.Cur: 54, 36 mA T.Cur/R.Cur = 67.5%, 45% Max.S/L: 120 m	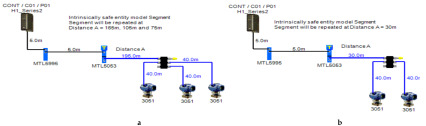
S/M: IS-E-TS Figure 7b No.S: a(2), b(2) No.T/S: a(3), b(2) P/S: MTL5995- MTL5053 A/V: 12.168 VDC C/U: 6 Port Brick T.Cur: 63, 42 mA T.Cur/R.Cur = 78.75%, 52.5% Max.S/L: 120 m	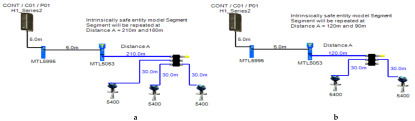
S/M: FISCO-IIB-DB Figure 7c No.S: a(2) No.T/S: a(7) P/S: MTL9122-IS A/V: 12.942 VDC C/U: 6 Port Brick T.Cur: 138 mA T.Cur/R.Cur = 53.9% Max.S/L: 60 m	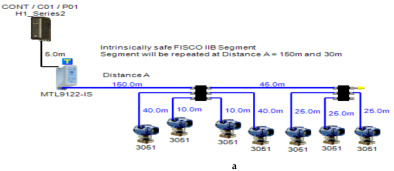
S/M: FISCO-IIB-TS Figure 7c No.S: a(1), b(1) No.T/S: a(8), b(2) P/S: MTL9122-IS A/V: 12.942 VDC C/U: 6 Port Brick T.Cur: 200, 54 mA T.Cur/R.Cur = 75.47%, 20.38% Max.S/L: 60 m	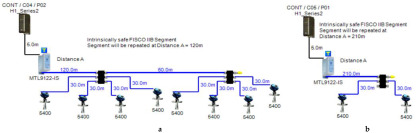
S/M: FISCO-IIC-DB Figure 7d No.S: a(3), b(1) No.T/S: a(4), b(2) P/S: MTL9121-IS A/V: 12.316 VDC C/U: 6 Port Brick T.Cur: 84, 68 mA T.Cur/R.Cur = 70%, 56.7% Max.S/L: 60 m	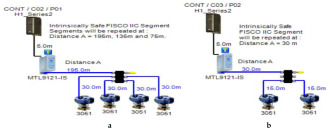
S/M: FISCO-IIC-TS Figure 7d No.S: a(2), b(1) No.T/S: a(4), b(2) P/S: MTL9121-IS A/V: 12.316 VDC C/U: 6 Port Brick T.Cur: 96, 54 mA T.Cur/R.Cur = 80%, 45% Max.S/L: 60 m	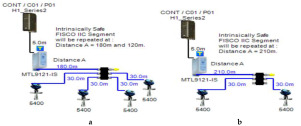
S/M: FISCO-IIB-DBSC Figure 7e No.S: a(2) No.T/S: a(7) P/S: MTL9122-IS A/V: 12.897 VDC C/U: JBBS-49SC-E413 T.Cur: 203 mA T.Cur/R.Cur = 76.6% Max.S/L: 60 m	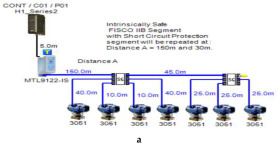
S/M: FISCO-IIB-TSSC Figure 7e No.S: a(1), b(1) No.T/S: a(6), b(4) P/S: MTL9122-IS A/V: 12.897,12.944 VDC C/U: JBBS-49SC-E413 T.Cur: 203, 156 mA T.Cur/R.Cur = 76.6%, 58.87%, Max.S/L: 60 m	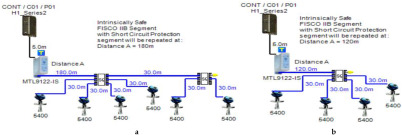
S/M: FNICO-IIB-DB Figure 7f No.S: a(1), b(1) No.T/S: a(12), b(2) P/S: MTL9112-IS-NI A/V: 12.852, 13.032 VDC C/U: 6 Port Brick T.Cur: 248, 68 mA T.Cur/R.Cur = 77.5%, 21.25% Max.S/L: 60 m	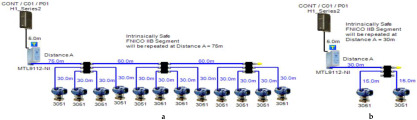
S/M: FNICO-IIB-TS Figure 7f No.S: a(1) No.T/S: a(10) P/S: MTL9112-NI A/V: 12.858 VDC C/U: 6 Port Brick T.Cur: 242 mA T.Cur/R.Cur = 75.63% Max.S/L: 60 m	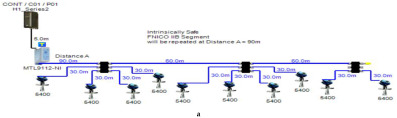
S/M: FNICO-IIB-DBSC Figure 7g No.S: a(1), b(1) No.T/S: a(8), b(6) P/S: MTL9112-NI A/V: 12.879, 12.915 VDC C/U: JBBS-49SC-E413 T.Cur: 221, 185 mA T.Cur/R.Cur = 69.1%, 57.81% Max.S/L: 60 m	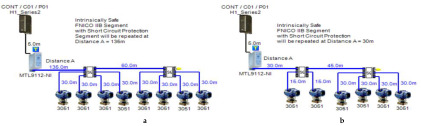
S/M: FNICO-IIB-TSSC Figure 7g No.S: a(1), b(1) No.T/S: a(7), b(3) P/S: MTL9112-NI A/V: 12.876, 12.965 VDC C/U: JBBS-49SC-E413 T.Cur: 224, 135 mA T.Cur/R.Cur = 70%, 42.19% Max.S/L: 60 m	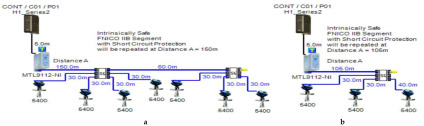
S/M: FNICO-IIC-DB Figure 7h No.S: a(2) No.T/S: a(7) P/S: MTL9111-NI A/V: 12.262 VDC C/U: 6 Port Brick T.Cur: 138 mA T.Cur/R.Cur = 76.7% Max.S/L: 60 m	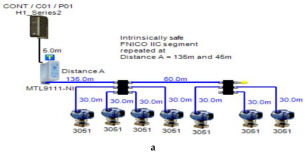
S/M: FNICO-IIC-TS Figure 7h No.S: a(2), b(1) No.T/S: a(4), b(2) P/S: MTL9111-NI A/V: 12.304, 12.346 VDC C/U: 6 Port Brick T.Cur: 96, 54 mA T.Cur/R.Cur = 53.33%, 30% Max.S/L: 60 m	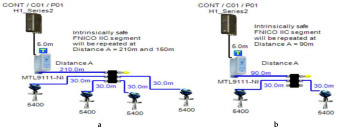
S/M: FNICO-IIC-DBSC Figure 7i No.S: a(4), b(1) No.T/S: a(3), b(2) P/S: MTL9111-NI A/V: 12.274, 12.292 VDC C/U: JBBS-49SC-E413 T.Cur: 126, 108 mA T.Cur/R.Cur = 70%, 60% Max.S/L: 60 m	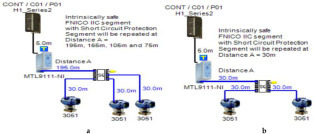
S/M: FNICO-IIC-TSSC Figure 7i No.S: a(2), b(2) No.T/S: a(3), b(2) P/S: MTL9111-NI A/V: 12.265, 12.286 VDC C/U: JBBS-49SC-E413 T.Cur: 135, 114 mA T.Cur/R.Cur = 75%, 63.33% Max.S/L: 60 m	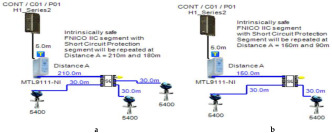
S/M: HPTC-SP-DB Figure 7a No.S: a(1) No.T/S: a(14) P/S: HD2-FBPS 1 2.3.500 A/V: 21 VDC C/U: R2-SP-N4 T.Cur: 338 mA T.Cur/R.Cur = 67.6% Max.S/L: 60 m	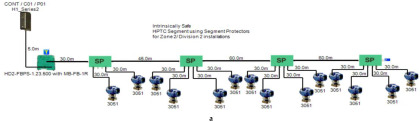
S/M: HPTC-SP-TS Figure 7a No.S: a(1) No.T/S: a(10) P/S: HD2-FBPS 1 2.3.500 A/V: 21 VDC C/U: R2-SP-N4 T.Cur: 292 mA T.Cur/R.Cur = 58.4% Max.S/L: 120 m	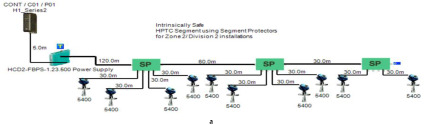
S/M: HPTC-FB-DB Figure 7a No.S: a(1) No.T/S: a(14) P/S: HD2-FBPS 1 2.3.500 A/V: 21 VDC C/U: FB-Ex4 T.Cur: 395.57 mA T.Cur/R.Cur = 79.114% Max.S/L: 60 m	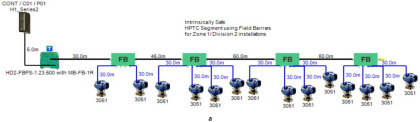
S/M: HPTC-FB-TS Figure 7a No.S: a(1) No.T/S: a(10) P/S: HD2-FBPS 1 2.3.500 A/V: 21 VDC C/U: FB-Ex4 T.Cur: 331.11 mA T.Cur/R.Cur = 66.222% Max.S/L: 120 m	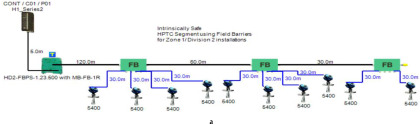
S/M: DART-DB Figure 7j No.S: a(1) No.T/S: a(10) P/S: HD2-FBPS-IBD-1.24.360 A/V: 21 VDC C/U: R3-SP-IBD12 T.Cur: 291 mA T.Cur/R.Cur = 80.83% Max.S/L: 60 m	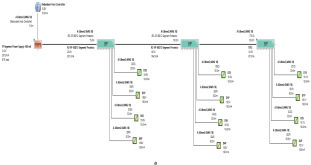
S/M: DART-DB Figure 7j No.S: b(1) No.T/S: b(2) P/S: HD2-FBPS-IBD-1.24.360 A/V: 21 VDC C/U: R3-SP-IBD12 T.Cur: 61 mA T.Cur/R.Cur = 16.95% Max.S/L: 120 m	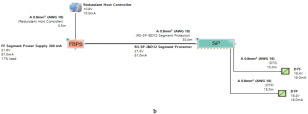
S/M: DART-TS Figure 7j No.S: a(1) No.T/S: a(10) P/S: HD2-FBPS-IBD-1.24.360 A/V: 21 VDC C/U: R3-SP-IBD12 T.Cur: 295 mA T.Cur/R.Cur = 81.95% Max.S/L: 90 m	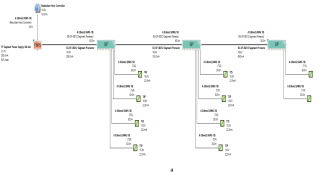

**Table 2:** Table indicating values of the coefficients for the polynomial Equation (10) defining the relation between the current consumed by the field barrier and H1 trunk cable length in each of the models illustrated in Figure 6a,b. Coefficients k_1_ in mA/$m^{4}$, k_2_ in mA/$m^{3}$, k_3_ in mA/$m^{2}$, k_4_ in mA/m and k_5_ in mA.

Model	Coefficients	Model	Coefficients
One Transmitter Rosemount 3051 $I_{\mathrm{dT}}$ = 18 mA	$k_{1}=$4.739$\times 10^{-15}$ $k_{2}$=−2.006$\times 10^{-11}$ $k_{3}=$1.928$\times 10^{-7}$ $k_{4}=$−0.0005017 $k_{5}=84.28$	One Transmitter Rosemount 5400 $I_{\mathrm{dT}}$ = 21 mA	$k_{1}=$1.537$\times 10^{-15}$ $k_{2}$=−1.202$\times 10^{-11}$ $k_{3}=$2.368$\times 10^{-7}$ $k_{4}=$−0.0004866 $k_{5}=86.4$
Two Transmitters Rosemount 3051 $I_{\mathrm{dT}}$ = 36 mA	$k_{1}=8.651\times 10^{-15}$ $k_{2}$=−1.425$\times 10^{-11}$ $k_{3}=$4.713$\times 10^{-7}$ $k_{4}=$0.0004212 $k_{5}=94.51$	Two Transmitters Rosemount 5400 $I_{\mathrm{dT}}$ = 42 mA	$k_{1}=$1.503$\times 10^{-14}$ $k_{2}$=−7.597$\times 10^{-13}$ $k_{3}=$1.045$\times 10^{-6}$ $k_{4}=$ −0.002513 $k_{5}=97.99$
Three Transmitter Rosemount 3051 $I_{\mathrm{dT}}$ = 54 mA	$k_{1}=$2.741$\times 10^{-14}$ $k_{2}$ = 2.596$\times 10^{-11}$ $k_{3}=$8.163$\times 10^{-7}$ $k_{4}=$0.001693 $k_{5}=105.9$	Three Transmters Rosemount 5400 $I_{\mathrm{dT}}$ = 63 mA	$k_{1}=$7.238$\times 10^{-14}$ $k_{2}$=−2.006$\times 10^{-11}$ $k_{3}=$ 1.928$\times 10^{-7}$ $k_{4}=$ −0.0005017 $k_{5}=111.345$
Four Transmitter Rosemount 3051 $I_{\mathrm{dT}}$ = 72 mA	$k_{1}=$ 1.723$\times 10^{-13}$ $k_{2}$ = −1.098$\times 10^{-10}$ $k_{3}=$ 1.282$\times 10^{-6}$ $k_{4}=$ 0.00359 $k_{5}=115.7$	Four Transmitters Rosemount 5400 $I_{\mathrm{dT}}$ = 84 mA	$k_{1}=$5.108$\times 10^{-13}$ $k_{2}$ = −6.964$\times 10^{-10}$ $k_{3}=$2.04$\times 10^{-6}$ $k_{4}=$ −0.004815 $k_{5}=122.4$

**Table 3:** Illustration of maximum allowable spur lengths in different FF models according to simulation results obtained from Emerson Segment Design Tool.

FF Model	Maximum Allowable Spur Length
Non-intrinsically safe	(1–10 field devices) (120 m) (11–12 field devices) (90 m) (Field devices > 12) (60 m)
HPTC	(1–11 field devices) (120 m) (12–13 field devices) (90 m) (Field devices > 13) (60 m)
DART	(1–10 field devices) (120 m) (11 devices) (90 m) (Field devices ≥ 13) (60 m)
Entity	120 m
FISCO	60 m
FNICO	60 m

## Data Availability

The data presented in this study are available on request from the corresponding author. The data are not publicly available due to concerns regarding possible exploitation of Simulink model and Matlab codes (Designed by corresponding author) without permission preserving the authors’ copy rights.

## Abbreviations

AMS	Asset Management System
CD	Compel Data
CSMA/CA	Carrier Sense Multiple Access/Collision Avoidance
DART	Dynamic Arc Recognition and Termination
DART DB	DART model for Double Bottom tanks
DART TS	DART model for Top Side tanks
DDT	Distributed Data Transfer
DSSS	Direct Sequence Spread Spectrum
FAS	Fieldbus Access Sublayer
FF	Foundation Fieldbus
FHSS	Frequency Hopping Spread Spectrum
FISCO	Fieldbus Intrinsically Safe Concept
FISCO-IIB-DB	FISCO IIB model for Double bottom ballast tanks without Short Circuit Protection
FISCO-IIB-DBSC	FISCO IIB model for Double bottom ballast Tanks with Short Circuit Protection
FISCO-IIB-TS	FISCO IIB model for Top Side ballast tanks without Short Circuit Protection
FISCO-IIB-TSSC	FISCO IIB model for Top Side ballast Tanks with Short-Circuit Protection
FISCO-IIC-DB	FISCO IIC model for Double bottom ballast tanks without Short Circuit Protection
FISCO-IIC-TS	FISCO IIC model for Top Side ballast tanks without Short Circuit Protection
FMS	Fieldbus Message Specification
FNICO	Fieldbus Non + Incendive Concept
FNICO-IIB-DB	FNICO IIB model for Double bottom ballast tanks without Short Circuit Protection
FNICO-IIB-DBSC	FNICO IIB model for Double bottom ballast Tanks with Short-Circuit Protection
FNICO-IIB-TS	FNICO IIB model for Top Side ballast tanks without Short Circuit Protection
FNICO-IIB-TSSC	FNICO IIB model for Top Side ballast Tanks with Short-Circuit Protection
FNICO-IIC-DBSC	FNICO IIC model for Double bottom ballast Tanks with Short-Circuit Protection
FNICO-IIC-DB	FNICO IIC model for Double bottom ballast tanks without Short Circuit Protection
FNICO-IIC-TS	FNICO IIB model for Top Side ballast tanks without Short Circuit Protection
FNICO-IIC-TSSC	FNICO IIC model for Top Side ballast Tanks with Short-Circuit Protection
HART	Highway Addressable Remote Transducer
HPTC-FB-DB	HPTC model with Field Barriers for Double Bottom tanks
HPTC-FB-TS	HPTC model with Field Barriers for Top Side tanks
HPTC-SP-DB	HPTC model with Segment Protectors for Double Bottom tanks
HPTC-SP-TS	HPTC model with Segment Protectors for Top Side tanks
HPTC	High-Power Trunk Concept
IEC	International Electrotechnical Committee
IS-E-DB	Intrinsically Safe Entity model for Double Bottom Tanks
IS-E-TS	Intrinsically Safe Entity model for Top Side Tanks
JRMNL	Joint Routing algorithm for maximizing network lifetime
LAS	Link Active Scheduler
LPF	Low-Pass Filter
LPG	Liquefied Petroleum Gas
MAC	Medium Access Control
NONIS-DB	Non-Intrinsically Safe model for Double Bottom Ballast Tanks without Short-Circuit Protection
NONIS-DBSC	Non-Intrinsically Safe model for Double Bottom Ballast Tanks with Short-Circuit Protection
NONIS-TS	Non-Intrinsically Safe model for Top Side Ballast Tanks without Short-Circuit Protection
NONIS-TS-SC	Non-Intrinsically Safe model for Top Side Ballast Tanks with Short-Circuit Protection
OSI	Open Systems Interconnection
PN	Probe Node
PT	Pass Token
SPM	Static Process Monitoring
SDT	Segment Design Tool
TD	Time Distribution
TDMA	Time Division Multiple Access
TSMP	Time Synchronization Mesh Protocol.
UART	Universal Asynchronous Receiver Transmitter

## Appendix A

### MATLAB Function in ADC in Simulink Model in Figure 4

The analogue-to-digital converter block in the Simulink model in Figure 4 consists of an idealized quantizer with $2^{n}$ possible digital outputs codes for an n-bit converter. The assumed analogue-to-digital converter is an 8-bit converter. MATLAB function ADC converts the quantized output value of the quantizer into an 8-bit binary value with MSB at the lower display and LSB at the upper display.

function [B1,B2,B3,B4,B5,B6,B7,B8] = ADC(u) gj = abs(u); ADCoutput = de2bi(gj,8,’right-msb’); B1 = ADCoutput(1); B2 = ADCoutput(2); B3 = ADCoutput(3); B4 = ADCoutput(4); B5 = ADCoutput(5); B6 = ADCoutput(6); B7 = ADCoutput(7); B8 = ADCoutput(8);

## Appendix B

### Datasheets

Power Supply HD2-FBPS 1 2.3.500	https://files.pepperl-fuchs.com/webcat/navi/productInfo/pds/255746_eng.pdf?v=20220326155821 (Last accessed on 1 August 2022)
Connection Megablock FCS-MB4	https://www.mtl-inst.com/images/uploads/datasheets/FCS-MBx%20Megablocks%20Rev4.pdf (Last accessed on 4 August 2022)
Connection Megablock JBBS-49SC-E413	TURCK Industrial Automation—Fieldbus Components For Foundation Fieldbus pp. 46–47 https://www.turck.de/att/D301024.pdf (Last accessed on 3 August 2022)
Isolated Power Supply MTL5995	https://www.mtl-inst.com/images/uploads/datasheets/5000/MTL5995.pdf (Last accessed on 7 August 2022)
Isolator/Power Supply MTL5053	https://www.mtl-inst.com/images/uploads/datasheets/EPS%20MTL5053.pdf (Last accessed on 1 August 2022)
FISCO IIB Power Supply MTL9122-IS	https://www.mtl-inst.com/images/uploads/datasheets/EPS%209122%20IS%20Rev%20G.pdf (Last accessed on 2 August 2022)
FISCO IIC Power Supply MTL9121-IS	https://www.mtl-inst.com/images/uploads/datasheets/EPS%209121%20IS%20Rev%20G.pdf (Last accessed on 2 August 2022)
FNICO IIB Power Supply MTL9112-NI	https://www.mtl-inst.com/images/uploads/datasheets/9112_NI.pdf (Last accessed on 6 August 2022)
FNICO IIC Power Supply MTL9111-NI	https://www.mtl-inst.com/images/uploads/datasheets/9111_NI.pdf (Last accessed on 1 August 2022)
Segment Protector R2-SP-N4	https://files.pepperl-fuchs.com/selector_files/navi/productInfo/doct/tdoct1234f_eng.pdf (Last accessed on 5 August 2022)
Field Barrier *-FB-Ex4.*	https://www.nexinstrument.com/assets/images/pdf/SPD0-FB-EX4.pdf (Last accessed on 8 August 2022)
DART Power Supply HD2-FBPS-IBD-1.24.360	https://files.pepperl-fuchs.com/webcat/navi/productInfo/pds/232465_eng.pdf?v=20171220000236 (Last accessed on 7 August 2022)
